# Shakespeare as a Physician

**Published:** 1884-08

**Authors:** 


SHAKESPEARE AS A PHYSICIAN, comprising every word which in any way relates to Medicine, Surgery or Obstetrics, found in the complete works of that writer, with criticisms and comparisons of the same with the medical thoughts of to-day. By J. PORTMAN CHESNEY, M.D., ex-Secretary Medical Society of State of Missouri, Corresponding member of the Geological Society of Boston; Professor of Gynæcology in the Northwestern College, St. Joseph, Mo., etc. J. H. Chambers & Co., Publishers, Chicago, St. Louis and Atlanta, 1884.

This work, while it does not embrace "every word which in any way relates to Medicine, Surgery or Obstetrics found in the complete works of that writer," it does contain a great deal that will prove of interest to the general reader, and especially to the physician.

